# Transcriptome Analysis Identifies Proteostasis and Cell Survival Pathway Disruption in Peripartum Cardiomyopathy, Leading to Heart Failure

**DOI:** 10.3390/cells15080698

**Published:** 2026-04-15

**Authors:** Pooja Choubey, Vanessa Montoya-Uribe, Michelle L. Matter

**Affiliations:** 1 The Lundquist Institute for Biomedical Innovation, Harbor-UCLA Medical Center, Torrance, CA 90502, USA; pooja.choubey@lundquist.org; 2 Department of Biochemistry and Molecular Biology, Tulane University School of Medicine, New Orleans, LA 70112, USA

**Keywords:** heart failure, apoptosis, peripartum cardiomyopathy, transcriptome, proteostasis, integrated stress response, mitochondria, cardiac remodeling

## Abstract

**Highlights:**

**What are the main findings?**

- RNA seq of human left ventricles with integrated ingenuity pathway analysis (IPA) defines a systems-level transcriptomic map of peripartum cardiomyopathy (PPCM), identifying 2891 genes shaping its molecular signature.
- Coordinated failure of proteostasis and translational control, driven by CLPP suppression and EIF2–LARP1 activation, reveals maladaptive stress remodeling.

**What are the implications of the main findings?**

- Disease-function and toxicity (IPA-TOX) analyses links transcriptional remodeling in PPCM to ventricular enlargement, fibrosis and cell death, mirroring PPCM echocardiographic features.
- Mechanistic modeling from IPA positions PPCM within the proteotoxic cardiomyopathy spectrum by identifying potential therapeutic targets in mitochondrial protease, integrated stress response (ISR), and COP9 pathways.

**Abstract:**

Peripartum cardiomyopathy (PPCM) is a pregnancy-associated form of systolic heart failure that develops when hemodynamic, metabolic, and hormonal stress of late gestation exceeds maternal cardiac adaptive capacity. While vascular, inflammatory, and genetic contributions have been implicated in PPCM, the integrated molecular programs connecting pregnancy-related stress to cardiomyocyte failure remain poorly defined. To elucidate these mechanisms, we performed a transcriptome-wide RNA seq of left ventricles from females with PPCM and non-failing female normal donor controls. Differential expression analysis identified 2891 genes with altered expressions (1491 upregulated, 1400 downregulated; fold change ≥ 2, FDR < 0.05). Ingenuity pathway analysis (IPA) revealed the activation of protein ubiquitination pathways, EIF2 signaling, mitochondrial dysfunction, and apoptosis pathways. Upstream regulator analysis indicated the suppression of mitochondrial protease *CLPP* (Z = −4.075) and activation of *COPS5* (Z = +5.982) and *TEAD1* (Z = +5.00), delineating dual regulatory modules of disease remodeling. Integrated network analysis demonstrated a loss of protein quality control and survival signaling with the activation of stress response and translational repression programs. This signifies a collapse of proteostasis and maladaptive adaptation. Collectively, these data define PPCM as a disorder of failed proteostasis and impaired translational homeostasis. Our analysis provides a systems-level framework connecting PPCM to ventricular dysfunction with potential therapeutic targets in mitochondria, protein quality-control, integrated stress–response, and COP9 signaling pathways.

## 1. Introduction

The link between pregnancy, the postpartum period, and heart failure was first described by Porak, in 1880 [1]. However, it was not until 1937 that the first well-documented clinical case of peripartum cardiomyopathy (PPCM) was reported in New Orleans, US establishing this disorder as a rare but life-threatening form of heart failure characterized by reduced ejection fraction (rEF) occurring in previously healthy women during late pregnancy or within five months postpartum [2,3,4]. PPCM is clinically defined by a reduction in left ventricular ejection fraction (LVEF) to below 45% in the absence of other identifiable causes of cardiomyopathy [4,5]. Although many affected individuals experience partial or complete recovery of cardiac function, a subset of patients progress to severe heart failure (HF), requiring mechanical circulatory support or cardiac transplantation if left untreated [6,7]. The incidence of PPCM varies widely depending on geographic and ethnic factors, with reported rates ranging from 1:300 to 1:4000 live births and a disproportionately higher prevalence in women of African and African American ancestry [8,9]. Because the genetic causes underlying PPCM are not fully defined, the condition continues to represent a significant cause of pregnancy-related cardiovascular morbidity and mortality. Currently, the clinical management of PPCM mainly relies on standard heart failure therapies used for other forms of structural heart diseases, specifically dilated cardiomyopathy (DCM) and HFrEF, including conventional neurohormonal antagonists such as beta-blockers, ACE inhibitors, and angiotensin receptor blockers (ARBs) [10,11]. These treatments aim to attenuate maladaptive remodeling and preserve cardiac function, with studies such as the IPAC study reporting the recovery of left ventricular ejection fraction to >50% in many patients within 12 months following diagnosis [12].

A healthy pregnancy inflicts profound mechanical stress on the heart [13,14] accompanied by the upregulation of adrenergic tone; renin–angiotensin–aldosterone system activation; and increased circulating levels of prolactin, estrogen, and progesterone [6,14]. These increased energetic demands are met by the maternal heart through the metabolic reprogramming of fatty acid oxidation and mitochondrial biogenesis [15]. In most females, these adaptations are well-tolerated via the activation of pro-survival kinases (*AKT*, *ERK*1/2), angiogenic signaling (*VEGF*, *PlGF*), and molecular chaperones that maintain proteostasis under high mechanical and oxidative stress [16,17]. However, a collapse of this reserve, gene mutations, or pathway dysregulation could lead to PPCM. Oxidative-stress-mediated cleavage of prolactin into anti-angiogenic 16 kDa fragments [18]; elevated circulating soluble fms-like tyrosine kinase-1 (s*Flt-1*) that sequesters *VEGF* and *PlGF* [6,7]; and dysregulated immune signaling with elevated interferon-gamma, *TNF-α*, and Fas-ligand [19,20] are some of the known factors of PPCM. Genetic studies demonstrate the enrichment of truncating variants in titin (*TTN*), desmoplakin (*DSP*) and lamin A (*LMNA*) [21,22] as a shared genetic susceptibility with DCM whereby pregnancy is the environmental stressor inducing PPCM [22,23]. Nevertheless, how these genetic mutations impact hemodynamic, hormonal, and immune changes during pregnancy to drive PPCM is not fully understood, and current models likely capture only part of a complex gene environment interplay [6,24].

To survive through mechanical and metabolic stresses, cardiomyocytes need a balanced and functional proteome. Protein quality-control (PQC) pathways including molecular chaperones (*HSP70*, *HSP90*, and small *HSP*s), the ubiquitin–proteasome system (UPS), autophagy–lysosome machinery, and mitochondrial proteases maintain cardiomyocyte proteostasis. Together, these systems preserve sarcomeric architecture, prevent the buildup of misfolded or oxidatively damaged proteins, and safeguard mitochondrial function. Any disruption of these mechanisms leads to proteotoxic stress, mitochondrial impairment, and impaired protein turnover, which can act as the key drivers of pathological cardiac remodeling and heart failure [16,19]. The integrated stress response (ISR) senses diverse stressors and activates eIF2α phosphorylation, leading to translational attenuation and selective translation of stress-adaptive transcription such as *ATF4*, *ATF3*, and *CHOP* [25,26]. In the heart, chronic ISR activation promotes maladaptive hypertrophy, cardiomyocyte apoptosis, and HF progression [27,28]. The failure of these systems leads to the accumulation of misfolded or aggregated proteins, triggering the activation of stress–response pathways including the unfolded protein response (UPR) in the endoplasmic reticulum (ER) and the mitochondrial unfolded protein response (UPRᵐᵗ) [29,30]. Single-cell and spatial transcriptomic studies of failing human hearts revealed the remodeling of ribosomal RNA processing, translation–initiation factors, and proteasomal subunits across cardiomyopathies [31,32]. These studies have largely focused on heterogeneous heart failure populations or non-PPCM cardiomyopathies, and therefore, disease-specific systems-level analyses remain limited [33].

Transcriptomic analyses of end-stage DCM hearts have identified overlapping metabolic and extracellular matrix remodeling pathways with PPCM [34,35]. A comprehensive, systems-level characterization of transcriptional programs related to proteostasis, mitochondrial quality control, translational stress, and pro-survival-death signaling has not been performed in human PPCM hearts. In this study, we performed a genome-wide transcriptional profiling of human PPCM left ventricular tissue and applied ingenuity pathway analysis (IPA)-based canonical pathway enrichment, upstream regulator prediction, regulator-effect network analysis, and cardiotoxicity profiling to define the molecular architecture of PPCM. By focusing on the interconnected stress–response modules, this work provides a systems-level framework for interpreting how PPCM impacts the transcriptional state and highlights candidate pathways for future functional and translational studies. This aims at improving risk stratification and enabling targeted interventions for pregnancy-associated HF.

## 2. Materials and Methods

### 2.1. Human Cardiac Tissue Procurement

De-identified formalin-fixed, paraffin-embedded (FFPE) left ventricular (LV) heart tissue from females with PPCM (*n* = 5) and non-failing female normal donor controls (*n* = 5) were obtained through Accio Biobank Online, via Corewell Health, MI, US, under Corewell Health IRB CHW 2017-198, the National Disease Research Interchange (NDRI) supported by NIH grant U42OD011158, and the Biorepository at the University of Hawaii John A. Burns School of Medicine (JABSOM). All samples were collected under institutional review board (IRB)-approved protocols in accordance with the Declaration of Helsinki. PPCM was diagnosed per the European Society of Cardiology criteria [3]: new-onset heart failure with LVEF < 45% during the last month of pregnancy or within five months postpartum, absence of other identifiable causes of HF, and absence of pre-existing cardiac disease. Normal donor control hearts had normal ventricular function and no history of cardiovascular disease.

### 2.2. RNA Extraction from FFPE Tissue

Total RNA was extracted from 80 µm FFPE LV tissue using the RecoverAll™ Total Nucleic Acid Isolation Kit (AM1975, Thermo Fisher Scientific, Waltham, MA, USA) following the manufacturer’s protocol. RNA quantity was measured using a Nano-Drop ND-1000 spectrophotometer (Thermo Fisher Scientific), and RNA integrity (RIN > 7.0) was assessed using an Agilent 2100 Bioanalyzer (Agilent Technologies, Santa Clara, CA, USA). Samples with an RNA concentration of >10 ng/µL and detectable 18S and 28S ribosomal peaks were included.

### 2.3. Human RNA Seq

RNA-seq library preparation and sequencing were performed by BGI Genomics (Cambridge, MA, USA) following the DNBSEQ-T7 protocols described previously [36]. Briefly, Poly-A mRNA was purified from 5 µg of total RNA using Dynabeads Oligo (dT; Thermo Fisher, Waltham, CA, USA) and fragmented at 94 °C. First-strand cDNA was synthesized via SuperScript II Reverse Transcriptase (1896649, Invitrogen, Carlsbad, CA, USA), followed by U-labeled second-strand synthesis (m0209, New England Biolabs, Ipswich, MA, USA). Libraries were prepared using the MGIEasy RNA Library Prep Set, including A-tailing, dual-index adapter ligation, and size selection (300 ± 50 bp) with AMPureXP beads. After UDG enzyme (m0280, New England Biolabs) treatment, libraries were amplified (8 cycles) and sequenced on the DNBSEQ-T7 platform (PE150). Bioinformatic analysis was done using IPA and pathway visualization was conducted using LC Sciences methodology and iDEP. Differential gene expression was defined by *p* < 0.05 and an absolute fold change of ≥1.5. Analysis focused on fifty DEGs associated with PPCM/DCM, cardiac hypertrophy, and conduction pathways.

### 2.4. Differential Gene Expression Analysis

Differentially expressed genes (DEGs) were identified using linear models with empirical Bayes moderation implemented in TAC. Multiple testing corrections were performed using the Benjamini–Hochberg false discovery rate (FDR) method. Genes with a fold change of ≥1.5 and FDR-adjusted *p* of <0.05 were considered significantly differentially expressed. Gene annotations were based on the NCBI Ref Seq and Ensembl databases (GRCh38/hg38). Inter-individual variability was present in the donor group and all samples were retained based on pre-specified quality criteria.

### 2.5. Ingenuity Pathway Analysis (IPA)

DEGs corresponding to protein-coding or multi-complex genes were uploaded into IPA software (QIAGEN Bioinformatics, Redwood City, CA, USA; Build:9, content version: 153384343) for core analysis. Pseudogenes, unassigned probes, and non-coding RNAs without functional annotation were excluded. IPA performed canonical pathway enrichment: Fisher’s exact test with Benjamini–Hochberg correction; pathways with *p* < 0.05 were considered significant. Activation Z-scores (Z > 2) predict activation or inhibition based on directional gene expression. Next, upstream regulator analysis predicted transcriptional regulators, cytokines, kinases, and other molecules responsible for the observed expression changes, using the overlap *p*-value (Fisher’s test) and activation Z-score. Regulator-effect networks connect upstream regulators to downstream disease/function annotations via intermediate molecules to predict causal flow. Disease and function annotation identified enriched biological functions and phenotypes using Fisher’s exact test and IPA-Tox analysis mapped DEGs to toxicity pathways relevant to cardiac pathology (cardiac hypertrophy, fibrosis, necrosis/cell death, heart failure). Statistical significance was set at *p* < 0.05 and Z-score > 2.0 where applicable.

### 2.6. Protein–Protein Interaction Map

An interaction map for proteins was generated using STRING (Version 12.0). The network view summarizes the predicted associations for groups of proteins. Default analysis provides calculations for the enrichment scores of *p*-values and FDR values.

### 2.7. iDEP v2.0

Data analysis for the enrichment maps and heatmaps was generated using iDEP v2.0. Default analysis provides calculations for the enrichment scores of *p*-values and FDR values. [37].

## 3. Results

### 3.1. Transcriptomic Profiling Reveals Segregation of PPCM and Normal Donor LV

**Figure 1:**
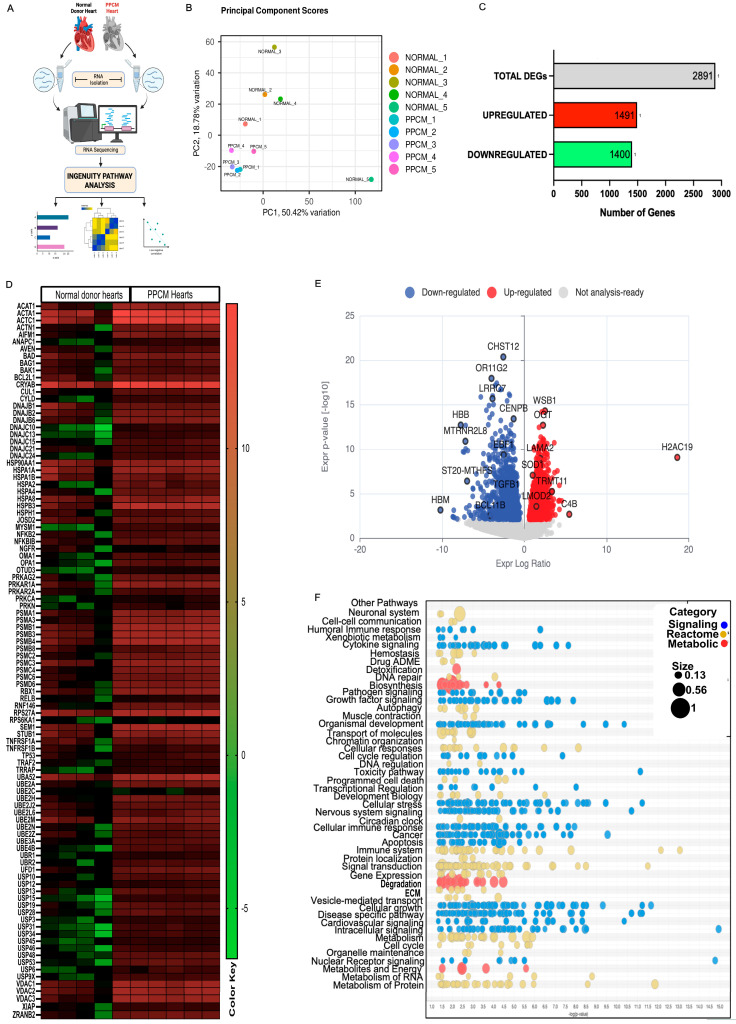
Global transcriptomic profiling of LVs in PPCM and normal donor samples: (**A**) Schematic overview of the experimental workflow. (**B**) Principal component analysis (PCA) of normalized expression for PPCM and control groups along PC1 (50.42%) and PC2 (18.76%) variance. (**C**) Bar graph summarizing DEG counts (absolute fold change ≥ 1.5, *p* < 0.05): 1491 upregulated (red) and 1400 downregulated (green) transcripts in PPCM. (**D**) Heatmap of representative proteostasis-related DEGs of HSP-family, chaperones, proteasome subunits and E3 ligases. Rows = genes (z-scored), columns = individual samples. (**E**) Volcano plot displaying log_2_ fold change (*x* axis) versus −log_10_ (*p*-value) (*y* axis). Genes with significant up (red)- or down (blue)-regulation are indicated; non-significant genes = gray. Labeled points denote transcripts. (**F**) Bubble volcano chart showing the distribution of canonical pathways as signaling (blue), reactome (golden) or metabolic pathways (red), with bubble size depicting the ratio of the number of overlapping genes to the total genes in the pathway. X axis = Z-score; Y axis = pathway category, with a display cutoff of −log (*p*-value) of 1.3. (**A**) was created in Biorender. Matter, M. (2026) https://BioRender.com/a09x4ib (accessed on 2 April 2026).

RNA seq of LV from PPCM patients (*n* = 5) and normal donor control (*n* = 5) was analyzed by IPA (Figure 1A). Further iDEP analyses yielded high quality normalized expression data across transcripts (Supplementary Figure S1A). Box-and-whisker plots of log_2_-transformed normalized intensities and density curves confirmed overlapping expression distributions and comparable signal ranges within each group (Supplementary Figure S1B,C). Principal component analysis (PCA) revealed a clear separation of PPCM and normal donor control samples along the first two principal components—PC1: 50.42% variance; PC2: 18.76% variance. One donor sample (NORMAL_5) exhibited greater separation along PC1 relative to the other control samples (Figure 1B), although this sample met all RNA quality and pairwise correlation thresholds (r ≥ 0.82) and was retained in the primary analysis. No samples exhibited globally low correlations indicative of technical failure. A total of 18,885 were identified as coding genes, 7 were Inc RNA and 2 were pseudogenes (Supplementary Figure S1D). Quality-control metrics demonstrated pairwise Pearson correlation coefficients of r  =  0.82–0.96 within groups, confirming intra-group correlation (Supplementary Figure S1F). Hierarchical clustering grouped all normal donor samples on one major branch and all PPCM samples on a separate branch, confirming robust transcriptional variation between the two conditions (Supplementary Figure S1E).

### 3.2. Differential Expression Analysis Identified 2891 Genes in PPCM Transcriptome

DEG analysis identified 2891 significantly dysregulated genes (fold change ≥ 1.5, *p* < 0.05) with 1491 upregulated and 1400 downregulated in PPCM left ventricles relative to donor controls (Figure 1C). Volcano plot (Figure 1E) analysis revealed genes with the largest effect sizes, including downregulated (*CHST12*, *OR11G2*, *LRRO7*, *TGFB*, *HBM*, *BCL11B*) and upregulated genes (*WSB1*, *OGT*, *LAMA2*, *SOD1*, *H2AC19*, *C4B*, *LMOD2*), indicating coordinated transcriptional changes associated with stress adaptation, metabolic activity, and structural remodeling. A heatmap of the top 100 differential genes identified proteostasis-related genes spanning chaperones (*HSP70/90*, *DNAJ*, *CRYAB*), ubiquitin conjugating enzymes (*UBE2A–UBE2Z* family), proteasome subunits (*PSMA*, *PSMB*, *PSMC*, *PSMD*), deubiquitinases (*USP* family and *ZRANB*), E3 ligases (*TRAF*), apoptosis regulators (*BAG*, *BAD*, *XIAP*), sarcomeric actins (*ACTA1*, *ACTC1*), and mitochondrial factors (*VDAC*) (Figure 1D). A coordinated upregulation of E2 enzymes, proteasome catalytic cores, and stress chaperones in PPCM, with selective downregulation of anti-apoptotic and mitochondrial genes, occurred across samples. A hierarchical k-means heatmap was across all samples (columns) for the coordinated up- (red) and downregulation (green) of all genes (rows; Supplementary Figure S1G) was produced. The regional enrichment analysis in iDEP utilized the sliding window approach via PREDA package to evaluate the ratio of DEGs relative to the total number of protein-coding genes within that specific genomic window. This chromosomal visualization function in iDEP identified six enriched regions on chromosomes 19 and 11. These segments represent contiguous chromosomal regions in which genes are coherently up- or downregulated, indicating that the transcriptional response is not randomly distributed but instead forms defined clusters. Such enrichment on chr19 and chr11 suggests potential locus level regulatory mechanisms, implying that genes within these regions may be co-regulated rather than acting independently (Supplementary Figure S1H).

### 3.3. Canonical Pathway Enrichment Reveals Dysregulation of Protein Ubiquitination, EIF2 Signaling, and Mitochondrial Function

**Figure 2:**
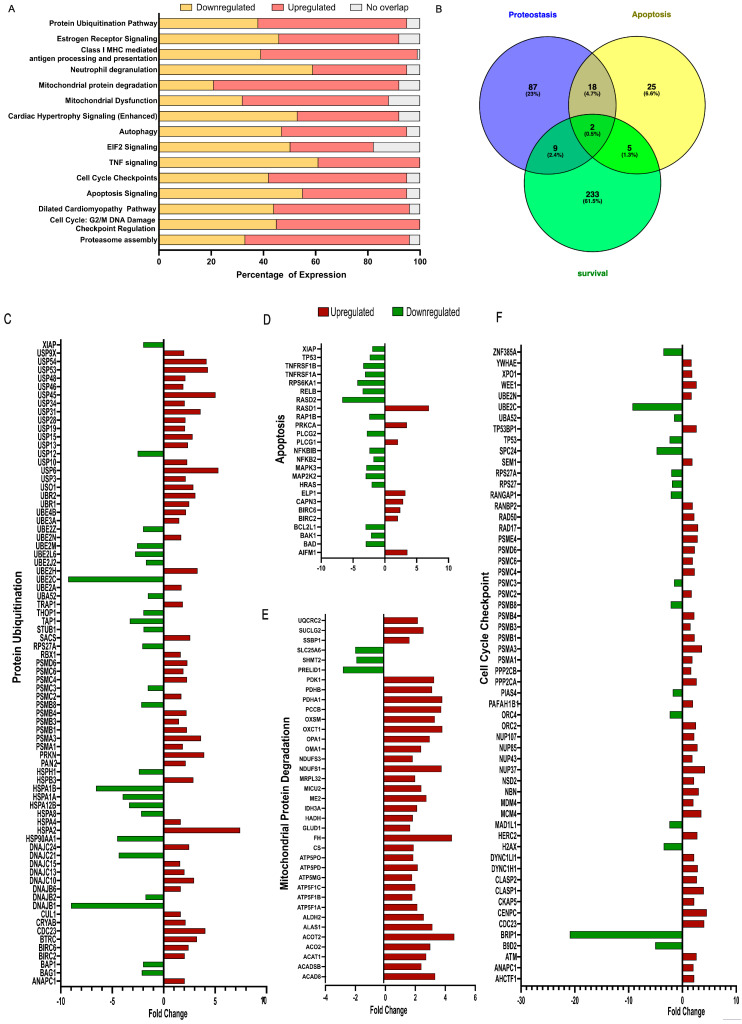
Canonical pathway enrichment identifies dysregulated proteostasis, mitochondrial quality control, and translational stress signaling. (**A**) Horizontal bar graph of top 15 canonical pathways ranked by −log_10_ (*p*-value). Pathway activation states inferred from IPA Z-scores: red bars = activated (Z ≥ 2), yellow bars = inhibited (Z ≤ −2). (**B**) Venn diagram of genes involved in proteostasis, apoptosis, and survival, highlighting the convergence between protein quality-control failure and programmed cell death. (**C**) Differential expression bar chart showing fold change in genes as up- (red) or downregulation (green) for protein ubiquitination, (**D**) apoptosis, (**E**) mitochondrial protein degradation and (**F**) cell-cycle checkpoint.

IPA identified a protein ubiquitination pathway as the top-most significantly enriched canonical pathway (*p* = 1.15 × 10^−15^), followed by estrogen receptor signaling (*p* = 1.7 × 10^−15^) and mitochondrial protein degradation (*p* = 1.44 × 10^−12^) (Figure 2A). Additional significantly enriched pathways included EIF2 signaling, autophagy, cardiac hypertrophy signaling, apoptosis signaling, TNF signaling, and JAK/STAT signaling (Figure 2A). Activation Z-scores indicated predicted inhibition of the mitochondrial dysfunction pathway (Z = −2.390) and activation of mitochondrial protein degradation (Z = +5.096), consistent with translational stress and metabolic remodeling. These results are summarized in a canonical pathway bubble volcano plot (Figure 1F), which categorizes pathways by biological function along the *Y* axis. By default, IPA assigns multiple functional groups, and a single pathway may be presented by multiple bubbles across different horizontal rows. Bubbles to a pathway are only displayed in a pathway category if it is significant. Each bubble represents one canonical pathway; the bubble color denotes the pathway class (blue = signaling, golden = reactome, red = metabolic); the bubble size reflects the gene ratio (overlapping DEGs/total pathway genes); and the *x*-axis position reflects the IPA activation Z-score. Pathway labels are displayed only for entries exceeding a −log(*p*-value) threshold of 1.3; pathways below this threshold are represented with no bubbles. The Venn diagram analysis (Figure 2B) details an overlap between genes annotated to proteostasis (233 genes), apoptosis (87 genes), and survival (25 genes), with 2 genes (*BAG1*, *STAT3*) shared across all three categories, consistent with the coordinated remodeling of stress–response programs, highlighting the convergence of protein quality-control failure and programmed cell death. Gene-level differential expression analyses across protein ubiquitination (Figure 2C), apoptosis (Figure 2D), mitochondrial protein degradation (Figure 2E), and cell-cycle checkpoint pathways (Figure 2F) revealed a marked upregulation of pro-apoptotic genes (*BAD*, *BAX*, *BID*, *APAF1*, *CASP3*, *CASP9*). In contrast, anti-apoptotic and DNA-repair genes (*BCL2*, *BRCA1*, *ATM*) displayed mixed or reduced expression, highlighting extensive transcriptional remodeling in PPCM left ventricles.

### 3.4. Upstream Regulator Analysis Identifies CLPP Inhibition and COPS5/TEAD1 Activation as Key Drivers of PPCM

**Figure 3:**
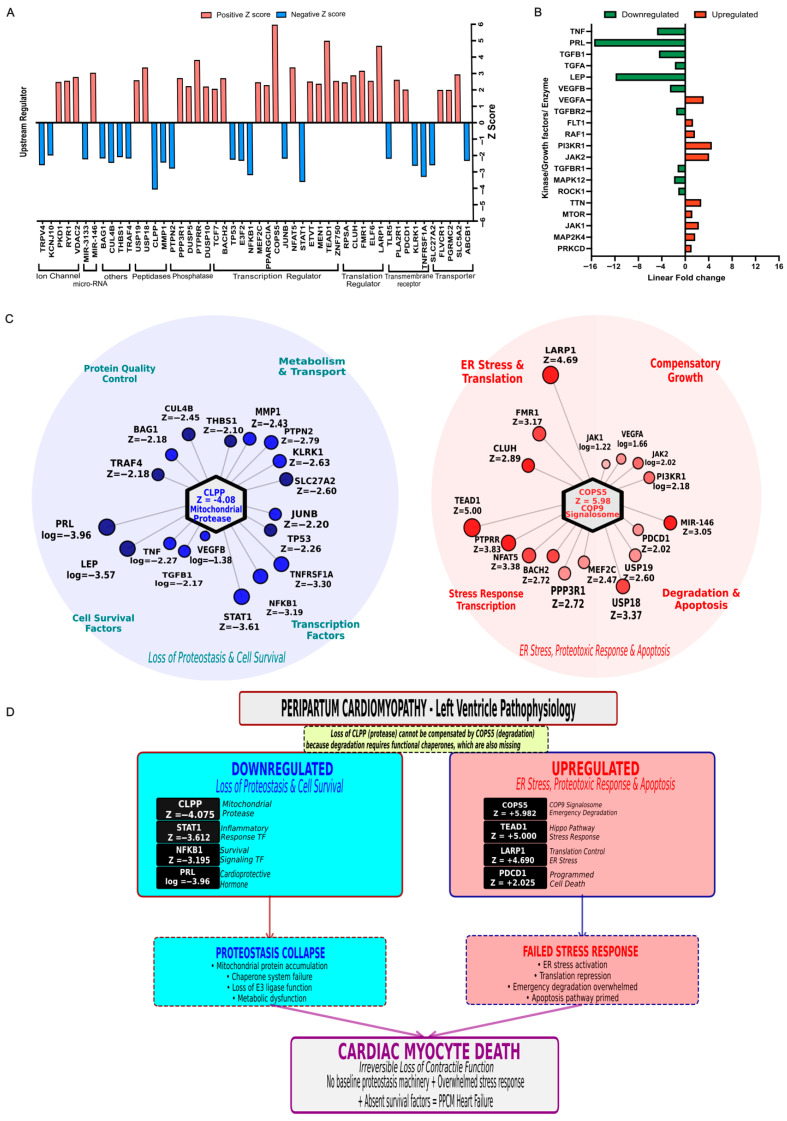
Upstream regulator analysis and integrated network model predict proteostasis collapse. (**A**) IPA upstream regulator analysis with predicted regulators by functional class (ion channels, microRNAs, peptidases, phosphatases, transcription regulators, translation regulators, transporters, and transmembrane receptors). Bars indicate Z-scores, with positive values (red) denoting predicted activation and negative values (blue) denoting predicted inhibition. (**B**) Expression of selected upstream regulators classified as kinases, growth factors, or enzymes are shown as linear fold change. Up- (red) and downregulated (green) regulators. (**C**) Network visualization of CLPP (left) and COP9 signalosome (right) as central upstream regulator hubs. The CLPP network displays the inhibition of protein quality-control factors, inflammatory mediators, and survival-associated transcription factors, with the loss of proteostasis and cell survival. The COP9 network indicates activated ER stress, translational control, compensatory growth, and apoptosis regulators, with protective stress response. Blue and red indicate inhibition and activation states and length of the nodes reflects the magnitude of the Z-score. (**D**) Schematic model summarizing the inhibition of CLPP (left) and activation of COP9 signalosome (right) to produce proteostasis collapse, failed stress adaptation, and cardiomyocyte death in PPCM.

Upstream regulator analysis in IPA identified key regulators including ion channels, peptidases, phosphatases, transcription regulators, transporters, and transmembrane receptors (Figure 3A). Positive Z-scores predicted the activation for *TNF*, *STAT3*, *NFKB1*, *TEAD1*, and *LARP1*, whereas PRL and LEP had negative Z-scores with predicted pathway inhibition. This pattern suggests the coordinated activation of stress- and growth-related signaling with concurrent inhibition of selected survival and hormonal pathways. The complementary expression analysis of enzymes, kinases, or growth factors (Figure 3B) predicted to be activated includes *COP9* signalosome components (*COPS5/COPS8*), *MAPK8*, *ROCK1*, and *PI3K* family members, which were upregulated at the mRNA level, whereas *TGFB1*, *TGFA*, and *PRL* were downregulated. This signaling shift suggests a remodeling of kinase and growth factor networks with stress and profibrotic activity. Based on IPA, two primary hubs, starting with the significant inhibition of the mitochondrial protease *CLPP* (Figure 3C; left), despite a modest individual transcript fold change, emerged as the most significantly inhibited upstream regulator (Z = −4.075, overlap *p* = 3.05 × 10^−7^), driven by the highly coordinated suppression of its predicted downstream network of 23 mitochondrial targets including fatty acid oxidation enzymes, TCA cycle components, and oxidative stress regulators whose collective upregulation is visualized in Supplementary Figure S2A and is consistent with substrate accumulation secondary to impaired proteolytic clearance. This triggers a cascade that inhibits essential quality-control and survival factors (*BAG1*, *STAT1*, *NFKB1*) and disrupts inflammatory and metabolic regulators. Ultimately, this convergence results in proteostasis collapse and reduced cell viability. The second hub (Figure 3C; right) indicates the activation of the *COP9* signalosome with Z = 5.98 and overlap *p* = 2.02 × 10^−8^ (Supplementary Figure S2), where this network triggers ER stress and activates key transcription factors such as *TEAD1*, *LARP1*, and *FMR1* that regulate compensatory growth and apoptosis.

Our findings link *CLPP* suppression (Figure 3D; left) and *COPS5* activation (Figure 3D; right) to a coordinated collapse of mitochondrial proteostasis and ubiquitin signaling. This is consistent with the contractile dysfunction and cardiomyocyte stress characteristic of end-stage PPCM, although the causal nature of these shifts remains to be confirmed through protein-level, functional, and in vivo mechanistic experiments. This transcriptome analysis highlights the *CLPP–COPS5* axis as a central candidate for investigating the mechanisms of irreversible heart failure in PPCM.

### 3.5. IPA Disease Predictions Indicate Multiple Dysregulated Pathways Creating an Imbalance Leading to HF

**Figure 4:**
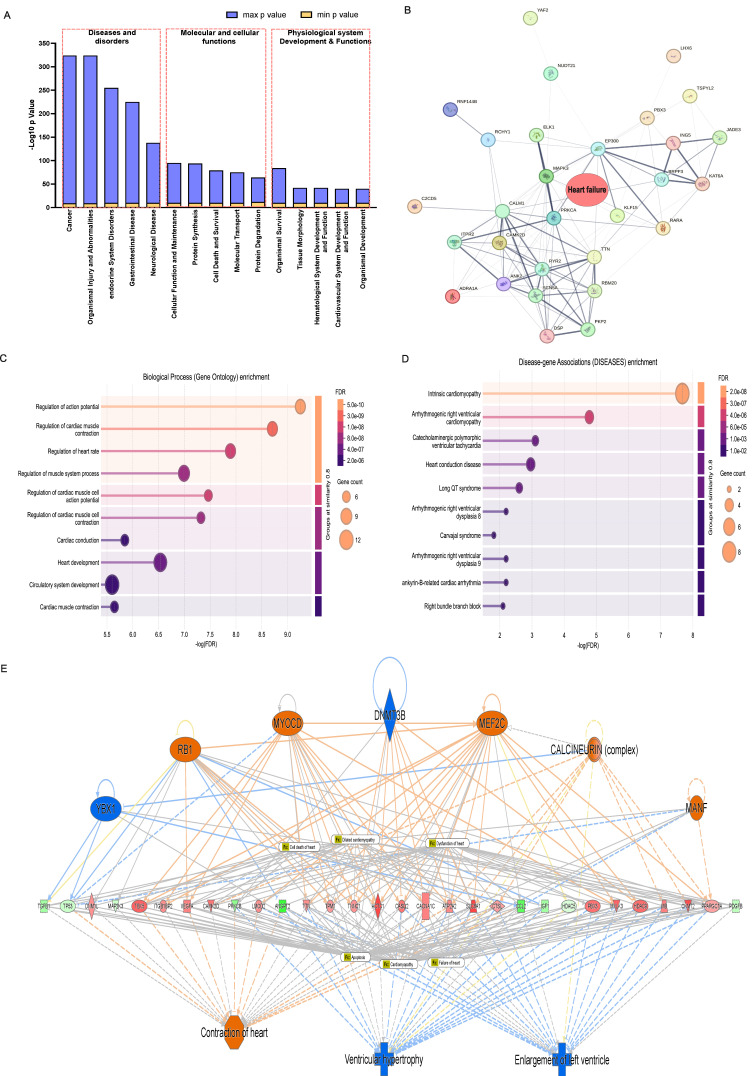
Disease and function level analyses reveals altered cell death and survival signaling in PPCM. (**A**) IPA Diseases and Function bars represent −log_10_ (*p*-value) for enriched functional categories; numbers indicate total associated molecules. Strong enrichment in molecular function and diseases was cell death/survival (*p* = 8.9 × 10^−44^*)*, protein synthesis (*p* = 1.2 × 10^−38^), and cardiac fibrosis (*p* = 5.0 × 10^−20^). (**B**) STRING protein–protein interaction network of heart-failure-disease-associated genes shows dense connectivity among stress signaling and mitochondrial quality-control hubs. (**C**,**D**) Disease and function enrichment plot confirming significant overrepresentation of heart failure, cardiomyopathy, and hypertrophy signatures. (**E**) Mechanistic model integrating IPA pathway predictions of cardiac-specific annotations, cardiac enlargement, dilation, fibrosis, and HF derived from IPA-Tox. Node color orange (activation) and blue (inhibition) intensity corresponds to statistical significance; edge thickness corresponds to interaction confidence.

The analysis of diseases, molecular and cellular functions, and physiological system development and functions revealed significant positive Z-scores across multiple categories (Figure 4A). Pathways associated with cell death; necrosis; and cardiac hypertrophy showed strong predicted activation, while cell survival; DNA repair; and protein synthesis pathways exhibited significant predicted inhibition. The protein–protein interaction network for heart failure-associated genes (Figure 4B) demonstrated connectivity among differentially expressed targets, with prominent hubs at stress-responsive transcription factors and mitochondrial quality-control factors, confirming the coordinated remodeling of heart failure-related protein networks. Disease enrichment (Figure 4C,D) displayed a significant overrepresentation of heart failure, dilated cardiomyopathy, and cardiac hypertrophy signatures among DEGs, with highly significant adjusted *p*-values (<10^−10^) and positive enrichment scores indicating the upregulation of disease-associated pathways.

IPA’s mechanistic disease prediction model (Figure 4E) illustrates the coordinated inhibition of cell survival and proteostasis pathways converging with hyperactivation of cell death, necrosis, and hypertrophy signaling to drive the maladaptive cardiac remodeling characteristic of PPCM.

## 4. Discussion

This study provides a comprehensive, systems-level transcriptional characterization of females with peripartum cardiomyopathy (PPCM). By integrating differential gene expression analysis using ingenuity pathway analysis (IPA) along with pathway enrichment, upstream regulator inference, and causal network modeling, we demonstrate that PPCM is associated with widespread dysregulation of multiple molecular pathways. This is characterized by the coordinated impairment of protein quality-control mechanisms, disruption of mitochondrial proteostasis, activation of EIF2-linked translational stress responses, and dysregulation of cardiomyocyte survival and death signaling pathways. Together, these molecular perturbations provide a mechanistic framework linking pregnancy-associated physiological stressors to cardiomyocyte dysfunction and adverse ventricular remodeling. Collectively, our findings position PPCM within the broader spectrum of proteotoxic cardiomyopathies and identify disrupted proteostasis as a central contributor of disease pathogenesis.

Among the most significant findings of this study is the transcriptional suppression of CLPP, a mitochondrial matrix serine protease. CLPP constitutes the proteolytic core of the ClpXP protease complex and functions as a critical component of mitochondrial quality control within cardiomyocytes [38,39]. This protease plays a central role in maintaining OXPHOS complex integrity by degrading misfolded or damaged respiratory chain subunits. Upon loss of CLPP in cardiomyocytes, the enzymatic activity of respiratory super complexes, particularly complexes I, III, and IV in cardiac mitochondria, is reduced [40]. Genetic deletion of CLPP in cardiac diseased murine model alleviates cardiomyopathy by reducing aberrant substrate turnover and restoring de novo OXPHOS synthesis [40]. However, CLPP has been reported to be moderately upregulated in the human heart as a stress-responsive mitochondrial protease, particularly in cardiomyocytes affected by Friedreich’s ataxia, where its expression is associated with mitochondrial iron–sulfur protein loss [41]. The dysregulation of CLPP has been proposed to contribute to cardiovascular pathology through impairing mitochondrial ATP production, disrupting ROS balance, and disrupting mitochondrial calcium signaling, thereby compromising cardiomyocyte metabolic and functional integrity [38]. These findings suggest that it is not the absence of CLPP, but rather its dysregulation relative to substrate load, that determines cardiac outcomes. In the context of PPCM, we observe the transcriptional downregulation of CLPP rather than substrate overproduction; therefore, such a deficit is predicted to impair the clearance of damaged OXPHOS subunits, compromise proper respiratory chain assembly, reduce mitochondrial ATP availability, and thereby exacerbate the bioenergetic crisis experienced by postpartum cardiomyocytes [38,39]. Hence, the observed downregulation of CLPP expression could be interpreted as a failure of mitochondrial proteostatis impacting mitochondrial protein quality control under conditions of physiological stress [39]. Another study in humans showed that the biallelic loss-of-function mutations in CLPP can cause Perrault syndrome type 3, a disorder characterized by sensorineural hearing loss and ovarian failure [42]. Whether affected individuals also exhibit cardiovascular manifestations remains unclear and is worth further investigation. The predicted consequence of *CLPP* downregulation causes an unchecked accumulation of damaged mitochondrial proteins, impaired UPRmt adaptation, and bioenergetic failure, which is mechanistically consistent with the cardiomyocyte death pathways identified across the broader transcriptomic dataset. The loss of *CLPP*-mediated proteolytic clearance is therefore expected to result in the accumulation of damaged and misassembled mitochondrial proteins, causing mitochondrial proteotoxic stress that activates the UPRmt, driven by transcription factors such as ATF5 and CHOP, which function to re-balance mitochondrial proteostasis [29,30]. We observed the coordinated upregulation of ATF3, ATF4, and DDIT3 (CHOP), together with the significant activation of EIF2 signaling (Z = +2.83), aligning with concurrent engagement of the integrated stress response downstream of mitochondrial proteotoxic and ER stress signals [25,26]. However, while transient UPRmt and ISR induction is pro-survival, sustained activation can become detrimental, driving apoptosis through the CHOP-dependent transcription of pro-apoptotic BH3-only proteins. These proteins promote mitochondrial-mediated apoptosis by directly activating BAX/BAK or by blocking anti-apoptotic BCL2 family members, thereby amplifying cardiomyocyte death signaling [43,44].

The mitochondrial compartment is not the only subcellular organelle that has impaired proteostasis in PPCM. Concomitant downregulation of cytosolic chaperones (HSPA8, DNAJA1) and proteasome subunits indicates a broader failure of the protein quality-control (PQC) network. This extends to endoplasmic reticulum and other cytosolic pathways that align with proteomic analyses of studies in end-stage DCM. In these studies, it was reported that protein aggregation and the accumulation of ubiquitin–protein conjugates at levels up to five-fold than those observed in healthy donor tissue are in part attributable to the oxidative modification of 19S ATPase subunits that impairs 26S proteasome proteolytic activity despite stable proteasomal subunit expression [45,46]. Proteomic analyses further distinguished PPCM from DCM by identifying unique disease-specific protein signatures, including the selective downregulation of SBSPON and TNS3 in PPCM. However, the depletion of sarcomeric proteins such as MYBPC3 and BAG3 and the persistent intrasarcoplasmic amyloid-like aggregates are shared characteristic hallmarks of end-stage failure across both etiologies [47]. Collectively, these findings underscore that the transition to HF in PPCM is heavily mediated by an overwhelmed UPS, highlighting restoration of proteostasis as a critical therapeutic target for mitigating disease progression [16].

In our study, EIF2 signaling was identified as the second-most enriched pathway (*p* = 3.98 × 10^−15^) in cardiomyocytes. The integrated stress response (ISR) is activated by four stress-sensing kinases: PERK (ER stress), GCN2 (amino acid deprivation), HRI (heme deficiency), and PKR (viral infection). These kinases converge on the phosphorylation of eIF2α at Ser51, resulting in the global suppression of protein translation while promoting the translation of stress-adaptive mRNAs such as *ATF4*, *ATF3*, *CHOP*, and *GADD34* [48,49]. Although transient ISR activation is protective, prolonged eIF2α phosphorylation disrupts protein synthesis, diminishes sarcomeric protein turnover, and ultimately promotes apoptosis in cardiomyocytes [25,50]. Complementary studies further show that the deacetylation of eIF2α by SIRT1 serves as a counter-regulatory mechanism that mitigates ER-stress-induced injury. Specifically, SIRT1 interacts with and deacetylates eIF2α, limiting the pro-apoptotic effects of the PERK pathway in cardiomyocytes [51]. This protective axis is further extended by the SIRT1-mediated activation of the eEF2K/eEF2 signaling pathway, which promotes adaptive autophagy and mitophagy, facilitating the clearance of damaged mitochondria during severe ER stress [52]. Supporting the therapeutic potential of this mechanism, the pharmacological activation of SIRT1 using natural polyphenols such as ferulic acid, pterostilbene, and tyrosol has been shown to reduce eIF2α-driven apoptosis and preserve cardiac function in vivo [53]. Our analysis of upstream regulators further identified *LARP1* (Z = +4.690) as a key activated regulatory node. Under stress conditions, *LARP1* acts as a molecular switch that sequesters 5′ terminal oligopyrimidine (TOP) mRNAs, which encode ribosomal proteins and elongation factors into inactive ribonucleoprotein complexes [54,55]. By preventing these mRNAs from reaching the ribosome, *LARP1* upregulation reinforces the translational repression initiated by eIF2α-phosphorylation. This creates a dual translational blockade where eIF2α phosphorylation inhibits the initiation of general protein synthesis, while *LARP1* specifically depletes the availability of the translational machinery itself. Together, these mechanisms may contribute to the severe contractile protein insufficiency characteristic of PPCM that exacerbates cardiomyocyte dysfunction.

Notably, *COPS5* (*COP9* signalosome subunit 5; also known as JAB1/CSN5) was the most-activated upstream regulator (Z = +5.982). COPS5 is a metalloprotease that removes NEDD8 from cullins, thereby regulating cullin-RING E3 ubiquitin ligase (CRL) activity [56,57]. CRLs control turnover of the cell-cycle, transcription, and signaling proteins, and COPS5 overexpression has been linked to cardiovascular disease, cancer, and inflammation [58]. In PPCM, COPS5 activation may dysregulate E3 ligase specificity, leading to aberrant degradation of pro-survival factors, including HIF1α and p53, and the accumulation of pro-apoptotic substrates. Contrary to models theorizing dominant apoptotic activation, our data suggests a simultaneous upregulation of pro-survival and pro-apoptotic genes, negating a uniform pro-death active state. Pro-survival kinases (*AKT1*, *PI3K*, *STAT3*) and anti-apoptotic BCL-2 family members (*BCL2*, *BCL2L1*) were upregulated alongside pro-apoptotic factors (*BAX*, *BAD*, *BID*, *PUMA*, *CASP3*, *CASP9*). This pattern is consistent with the chronic mechanical and oxidative stress signaling seen in PPCM, in which cardiomyocytes attempt to maintain cellular viability via compensatory AKT/mTOR activation but remain ‘primed’ for apoptosis [59,60]. This response likely brings a surge of PPCM-specific stressors such as the anti-angiogenic and pro-apoptotic 16 kDa prolactin fragment and the late-pregnancy hemodynamic together to exhaust the heart’s compensatory capacity [6,18]. The dual activation of both pro-survival and pro-apoptotic pathways may help explain the clinical heterogeneity observed in PPCM where some patients resolve hormonal and inflammatory stress specifically by neutralizing the cardiotoxic effects of prolactin, showing recovery in ventricular function while others with persistent stressors progress to HF. This mechanism is supported by clinical evidence showing that early bromocriptine therapy, a dopamine agonist that blocks prolactin secretion, prevents the downstream generation of oxidative stress, resulting in significant improvement in LVEF recovery in patients [61,62].

Based on our analysis and the existing literature, we conceptualize PPCM pathogenesis as arising from four interconnected pathogenic mechanisms: (1) a genetic–environmental hit, in which variants in sarcomeric genes such as *TTN* reduce structural cardiac stability [29]; (2) oxidative-stress-driven cleavage of prolactin generating the cardiotoxic 16 kDa prolactin fragment, a process often linked to *STAT3* deficiency [18]; (3) an angiogenic–metabolic imbalance characterized by elevated sFlt-1 and suppressed VEGF/PlGF signaling [6,7] and (4) proteostatic failure where the loss of molecular safeguards leads to progressive cellular dysfunction and collapse [6]. Recent studies indicate that a substantial proportion of PPCM patients lack identifiable mutations in known structural cardiomyopathy genes, underscoring the need to define the disease’s post-transcriptional and proteostatic landscape [21,63]. However, it is important to note that there have been only a handful of studies analyzing genetic changes in PPCM patients and even fewer studies that have used WES approaches to identify pathogenic variants.

Experimental evidence from our group provides independent support for the centrality of proteostatic mechanisms in PPCM. Montoya-Uribe et al. recently demonstrated that the cardiac-specific deletion of *Ptrh2* in female mice recapitulates key features of PPCM including enhanced cardiomyocytes apoptosis and progressive cardiac dysfunction [36]. Notably, the transcriptional dysregulation of proteostatic and translational stress pathways observed in human PPCM LVs in the present study, including the suppression of mitochondrial proteases, activation of the ISR, and impairment of cytosolic PQC, are broadly consistent with the molecular and cellular phenotypes observed in this experimental PPCM mouse model.

**Figure 5:**
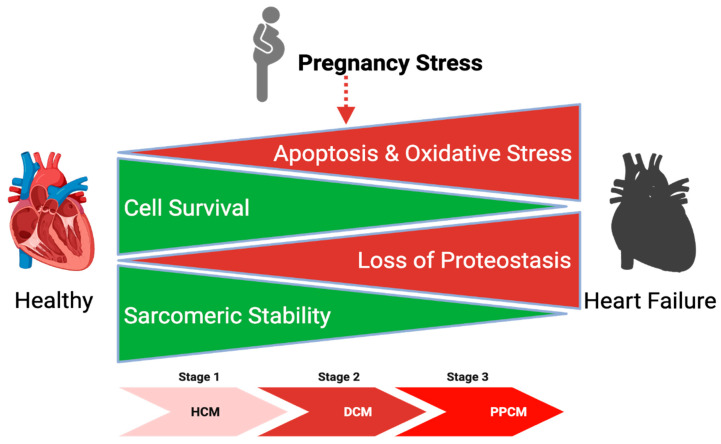
Proposed two-phase model of PPCM pathogenesis. Pregnancy-associated physiological stress shifts cardiac homeostasis toward a pathological state characterized by increased apoptosis and oxidative stress (top, red) together with impaired proteostasis (bottom, red). These molecular disturbances reduce cardiomyocyte survival and compromise sarcomeric stability, thereby promoting progressive myocardial remodeling. Overtime, this process facilitates the transition from compensatory hypertrophy to dilated cardiomyopathy, ultimately contributing to the development of peripartum cardiomyopathy. Figure was created in BioRender. Matter, M. (2026) https://BioRender.com/98pzrlx (accessed on 2 April 2026).

Integrating these findings, we propose a two-phase model of PPCM pathogenesis (Figure 5). In Phase 1, pregnancy-induced hemodynamic and hormonal stressors activate adaptive cardioprotective pathways including proteostasis maintenance, angiogenic signaling, and metabolic remodeling. In most individuals, these responses are sufficient to promote compensatory hypertrophy and preserve cardiac homeostasis during pregnancy. In Phase 2, however, susceptible pregnant women—such as those with hypertensive disorders of pregnancy, pathogenic gene mutations previously associated with PPCM or yet-unidentified variants affecting proteostasis-related pathways—possess a limited baseline PQC capacity that is inadequate to meet the increased physiological demands. The resulting progressive imbalance between survival and apoptotic signaling promotes hypertrophic remodeling, ultimately transitioning to a dilated cardiomyopathy and, in severe cases, progression to end-stage heart failure.

## 5. Limitations

This study had several key limitations. Our sample size was constrained by the limited availability of human LV tissue; therefore, validation in larger and independent cohorts will be necessary to confirm these findings and to better characterize heterogeneity among PPCM subtypes (e.g., early vs. late onset and recovered vs. persistent dysfunction). In addition, future studies utilizing single-cell RNA sequencing or spatial transcriptomic approaches may overcome the limitations inherent to bulk tissue analyses by enabling cell-type-specific resolution of gene expression patterns. Furthermore, because mRNA abundance does not necessarily correlate with protein levels or post-translational modifications, complementary proteomic studies will be required to validate and extend the transcriptomic observations reported here. Finally, our analysis does not identify potential genetic variants that may contribute to PPCM pathogenesis. Comprehensive genomic studies, such as whole-exome sequencing in larger PPCM cohorts, will be essential to identify mutations in genes that regulate the exacerbated mechanical stresses associated with pregnancy and that may predispose individuals to the development of PPCM.

## 6. Conclusions

The PPCM heart exhibits a transcriptional signature characterized, in part, by impaired proteostasis evidenced by *CLPP* suppression and molecular chaperone loss. These alterations occur concomitantly with translational stress signaling mediated by *EIF2/LARP1* activation and de-neddylation-mediated dysregulation of ubiquitin ligases via COPS5 activation. Collectively, these molecular pathways converge to reprogram cardiomyocyte survival and death signaling networks, thereby promoting the development of distinct cardiotoxic phenotypes. Specifically, within this proteotoxic environment, cardiomyocytes appear to transition from an initial compensatory hypertrophy state toward a dilated cardiomyopathy-like phenotype, characterized by left ventricular chamber dilation, interstitial fibrosis, and global contractile dysfunction. By positioning PPCM within the broader spectrum of proteotoxic cardiomyopathies, our systems-level framework identifies proteostasis reinforcement—potentially through modulation or inhibition of integrated stress–response mediators—as a plausible therapeutic strategy to mitigate disease progression and promote ventricular functional recovery.

## Data Availability

The original contributions presented in this study are included in the article/Supplementary Materials. Further inquiries can be directed to the corresponding author(s).

## Supplementary Materials

The following supporting information can be downloaded at https://www.mdpi.com/article/10.3390/cells15080698/s1. Figure S1: Quality control metrics and genome wide expression distributions. Figure S2: Network visualization of upstream regulators by IPA, Source data.

## References

[1] Porak C. (1880). De L’influence Réciproque de la Grossesse et des Maladies du Coeur.

[2] Hull E., Hafkesbring E. (1937). Toxic postpartal heart disease. New Orleans Med. Surg. J..

[3] Sliwa K., Hilfiker-Kleiner D., Petrie M.C., Mebazaa A., Pieske B., Buchmann E., Regitz-Zagrosek V., Schaufelberger M., Tavazzi L., van Veldhuisen D.J. (2010). Current state of knowledge on aetiology, diagnosis, management, and therapy of peripartum cardiomyopathy: A position statement from the Heart Failure Association of the European Society of Cardiology Working Group on peripartum cardiomyopathy. Eur. J. Heart Fail..

[4] Pearson G.D., Veille J.-C., Rahimtoola S., Hsia J., Oakley C.M., Hosenpud J.D., Ansari A., Baughman K.L. (2000). Peripartum cardiomyopathy: National heart, lung, and blood institute and office of rare diseases workshop recommendations and review. JAMA.

[5] Hibbard J.U., Lindheimer M., Lang R.M. (1999). A modified definition for peripartum cardiomyopathy and prognosis based on echocardiography. Obstet. Gynecol..

[6] Bauersachs J., König T., van der Meer P., Petrie M.C., Hilfiker-Kleiner D., Mbakwem A., Hamdan R., Jackson A.M., Forsyth P., de Boer R.A. (2019). Pathophysiology, diagnosis and management of peripartum cardiomyopathy: A position statement from the Heart Failure Association of the European Society of Cardiology Study Group on peripartum cardiomyopathy. Eur. J. Heart Fail..

[7] Arany Z., Elkayam U. (2016). Peripartum Cardiomyopathy. Circulation.

[8] Petersen E.E., Davis N.L., Goodman D., Cox S., Syverson C., Seed K., Shapiro-Mendoza C., Callaghan W.M., Barfield W. (2019). Racial/Ethnic Disparities in Pregnancy-Related Deaths—United States, 2007–2016. MMWR-Morb. Mortal. Wkly. Rep..

[9] Sliwa K., Mebazaa A., Hilfiker-Kleiner D., Petrie M.C., Maggioni A.P., Laroche C., Regitz-Zagrosek V., Schaufelberger M., Tavazzi L., van der Meer P. (2017). Clinical characteristics of patients from the worldwide registry on peripartum cardiomyopathy (PPCM). Eur. J. Heart Fail..

[10] Heidenreich P.A., Bozkurt B., Aguilar D., Allen L.A., Byun J.J., Colvin M.M., Deswal A., Drazner M.H., Dunlay S.M., Evers L.R. (2022). 2022 AHA/ACC/HFSA Guideline for the Management of Heart Failure. Circulation.

[11] McDonagh T.A., Metra M., Adamo M., Gardner R.S., Baumbach A., Böhm M., Burri H., Butler J., Čelutkienė J., Chioncel O. (2021). 2021 ESC Guidelines for the diagnosis and treatment of acute and chronic heart failure. Eur. Heart J..

[12] McNamara D.M., Elkayam U., Alharethi R., Damp J., Hsich E., Ewald G., Modi K., Alexis J.D., Ramani G.V., Semigran M.J. (2015). Clinical Outcomes for Peripartum Cardiomyopathy in North America: Results of the IPAC Study (Investigations of Pregnancy-Associated Cardiomyopathy). J. Am. Coll. Cardiol..

[13] Meah V.L., Cockcroft J.R., Backx K., Shave R., Stöhr E.J. (2016). Cardiac output and related haemodynamics during pregnancy: A series of meta-analyses. Heart.

[14] Rizi S.S., Wiens E., Hunt J., Ducas R. (2024). Cardiac physiology and pathophysiology in pregnancy. Can. J. Physiol. Pharmacol..

[15] Schulman-Geltzer E.B., Collins H.E., Hill B.G., Fulghum K.L. (2023). Coordinated Metabolic Responses Facilitate Cardiac Growth in Pregnancy and Exercise. Curr. Heart Fail. Rep..

[16] McLendon P.M., Robbins J. (2015). Proteotoxicity and Cardiac Dysfunction. Circ. Res..

[17] Wang X., Robbins J. (2006). Heart failure and protein quality control. Circ. Res..

[18] Hilfiker-Kleiner D., Kaminski K., Podewski E., Bonda T., Schaefer A., Sliwa K., Forster O., Quint A., Landmesser U., Doerries C. (2007). A cathepsin D-cleaved 16 kDa form of prolactin mediates postpartum cardiomyopathy. Cell.

[19] Sliwa K., Förster O., Libhaber E., Fett J.D., Sundstrom J.B., Hilfiker-Kleiner D., Ansari A.A. (2006). Peripartum cardiomyopathy: Inflammatory markers as predictors of outcome in 100 prospectively studied patients. Eur. Heart J..

[20] Sliwa K., Fett J., Elkayam U. (2006). Peripartum cardiomyopathy. Lancet.

[21] Goli R., Li J., Brandimarto J., Levine L.D., Riis V., McAfee Q., DePalma S., Haghighi A., Seidman J.G., Seidman C.E. (2021). Genetic and Phenotypic Landscape of Peripartum Cardiomyopathy. Circulation.

[22] Ware J.S., Li J., Mazaika E., Yasso C.M., DeSouza T., Cappola T.P., Tsai E.J., Hilfiker-Kleiner D., Kamiya C.A., Mazzarotto F. (2016). Shared genetic predisposition in peripartum and dilated cardiomyopathies. N. Engl. J. Med..

[23] Bozkurt B., Colvin M., Cook J., Cooper L.T., Deswal A., Fonarow G.C., Francis G.S., Lenihan D., Lewis E.F., McNamara D.M. (2016). Current Diagnostic and Treatment Strategies for Specific Dilated Cardiomyopathies: A Scientific Statement from the American Heart Association. Circulation.

[24] Davis M.B., Arany Z., McNamara D.M., Goland S., Elkayam U. (2020). Peripartum Cardiomyopathy: JACC State-of-the-Art Review. J. Am. Coll. Cardiol..

[25] Costa-Mattioli M., Walter P. (2020). The integrated stress response: From mechanism to disease. Science.

[26] Pakos-Zebrucka K., Koryga I., Mnich K., Ljujic M., Samali A., Gorman A.M. (2016). The integrated stress response. EMBO Rep..

[27] Santos-Ribeiro D., Godinas L., Pilette C., Perros F. (2018). The integrated stress response system in cardiovascular disease. Drug Discov. Today.

[28] Pu Y., Wu D., Lu X., Yang L. (2019). Effects of GCN2/eIF2α on myocardial ischemia/hypoxia reperfusion and myocardial cells injury. Am. J. Transl. Res..

[29] Shpilka T., Haynes C.M. (2018). The mitochondrial UPR: Mechanisms, physiological functions and implications in ageing. Nat. Rev. Mol. Cell Biol..

[30] Fiorese C.J., Schulz A.M., Lin Y.-F., Rosin N., Pellegrino M.W., Haynes C.M. (2016). The Transcription Factor ATF5 Mediates a Mammalian Mitochondrial UPR. Curr. Biol..

[31] Lee S.E., Joo J.H., Hwang H.S., Chen S.-F., Evans D., Lee K.Y., Kim K.-H., Hyun J., Kim M.-S., Jung S.-H. (2025). Spatial transcriptional landscape of human heart failure. Eur. Heart J..

[32] Chaffin M., Papangeli I., Simonson B., Akkad A.D., Hill M.C., Arduini A., Fleming S.J., Melanson M., Hayat S., Kost-Alimova M. (2022). Single-nucleus profiling of human dilated and hypertrophic cardiomyopathy. Nature.

[33] Wang L., Yu P., Zhou B., Song J., Li Z., Zhang M., Guo G., Wang Y., Chen X., Han L. (2020). Single-cell reconstruction of the adult human heart during heart failure and recovery reveals the cellular landscape underlying cardiac function. Nat. Cell Biol..

[34] Li A., Fang B., Li M., Koay Y.C., Malecki C., Hunter B., Harney D., Dos Remedios C.G., Larance M., O’Sullivan J.F. (2024). Myocardial posttranscriptional landscape in peripartum cardiomyopathy. Circ. Heart Fail..

[35] Taylor J., Yeung A.C., Ashton A., Faiz A., Guryev V., Fang B., Lal S., Grosser M., Dos Remedios C.G., Braet F. (2023). Transcriptomic comparison of human peripartum and dilated cardiomyopathy identifies differences in key disease pathways. J. Cardiovasc. Dev. Dis..

[36] Montoya-Uribe V., Choubey P., Walton C.B., Glibetic N., Seok Yang W., Aan F.J., Lindsey S., Peplowska K., Hernandez B.Y., Ramos J.W. (2025). Peptidyl-tRNA hydrolase 2 is a negative regulator of peripartum cardiomyopathy with heart failure in female mice. Nat. Commun..

[37] Ge S.X., Son E.W., Yao R. (2018). iDEP: An integrated web application for the differential expression and pathway analysis of RNA-Seq data. BMC Bioinform..

[38] Luo B., Ma Y., Zhou Y., Zhang N., Luo Y. (2021). Human ClpP protease, a promising therapy target for diseases of mitochondrial dysfunction. Drug Discov. Today.

[39] Chen Z., Huang L., Tso A., Wang S., Fang X., Ouyang K., Han Z. (2021). Mitochondrial chaperones and proteases in cardiomyocytes and heart failure. Front. Mol. Biosci..

[40] Seiferling D., Szczepanowska K., Becker C., Senft K., Hermans S., Maiti P., König T., Kukat A., Trifunovic A. (2016). Loss of CLPP alleviates mitochondrial cardiomyopathy without affecting the mammalian UPRmt. EMBO Rep..

[41] Bhandari V., Wong K.S., Zhou J.L., Mabanglo M.F., Batey R.A., Houry W.A. (2018). The role of ClpP protease in bacterial pathogenesis and human diseases. ACS Chem. Biol..

[42] Jenkinson E.M., Rehman A.U., Walsh T., Clayton-Smith J., Lee K., Morell R.J., Drummond M.C., Khan S.N., Naeem M.A., Rauf B. (2013). Perrault syndrome is caused by recessive mutations in CLPP, encoding a mitochondrial ATP-dependent chambered protease. Am. J. Hum. Genet..

[43] Li Y., Guo Y., Tang J., Jiang J., Chen Z. (2014). New insights into the roles of CHOP-induced apoptosis in ER stress. Acta Biochim. Biophys. Sin..

[44] Oyadomari S., Mori M. (2004). Roles of CHOP/GADD153 in endoplasmic reticulum stress. Cell Death Differ..

[45] Predmore J.M., Wang P., Davis F., Bartolone S., Westfall M.V., Dyke D.B., Pagani F., Powell S.R., Day S.M. (2010). Ubiquitin proteasome dysfunction in human hypertrophic and dilated cardiomyopathies. Circulation.

[46] Wang X., Li J., Zheng H., Su H., Powell S.R. (2011). Proteasome functional insufficiency in cardiac pathogenesis. Am. J. Physiol. Heart Circ. Physiol..

[47] Bollen I.A.E., Ehler E., Fleischanderl K., Bouwman F., Kempers L., Ricke-Hoch M., Hilfiker-Kleiner D., dos Remedios C.G., Krüger M., Vink A. (2017). Myofilament Remodeling and Function Is More Impaired in Peripartum Cardiomyopathy Compared with Dilated Cardiomyopathy and Ischemic Heart Disease. Am. J. Pathol..

[48] Harding H.P., Zhang Y., Zeng H., Novoa I., Lu P.D., Calfon M., Sadri N., Yun C., Popko B., Paules R. (2003). An integrated stress response regulates amino acid metabolism and resistance to oxidative stress. Mol. Cell.

[49] Matsui T., Tao J., Del Monte F., Lee K.-H., Li L., Picard M., Force T.L., Franke T.F., Hajjar R.J., Rosenzweig A. (2001). Akt activation preserves cardiac function and prevents injury after transient cardiac ischemia in vivo. Circulation.

[50] English A.M., Green K.M., Moon S.L. (2022). A (dis)integrated stress response: Genetic diseases of eIF2α regulators. Wiley Interdiscip. Rev. RNA.

[51] Prola A., Pires Da Silva J., Guilbert A., Lecru L., Piquereau J., Ribeiro M., Mateo P., Gressette M., Fortin D., Boursier C. (2017). SIRT1 protects the heart from ER stress-induced cell death through eIF2α deacetylation. Cell Death Differ..

[52] Pires Da Silva J., Monceaux K., Guilbert A., Gressette M., Piquereau J., Novotova M., Ventura-Clapier R., Garnier A., Lemaire C. (2020). SIRT1 Protects the Heart from ER Stress-Induced Injury by Promoting eEF2K/eEF2-Dependent Autophagy. Cells.

[53] Monceaux K., Gressette M., Karoui A., Pires Da Silva J., Piquereau J., Ventura-Clapier R., Garnier A., Mericskay M., Lemaire C. (2022). Ferulic Acid, Pterostilbene, and Tyrosol Protect the Heart from ER-Stress-Induced Injury by Activating SIRT1-Dependent Deacetylation of eIF2α. Int. J. Mol. Sci..

[54] Cassidy K.C., Lahr R.M., Kaminsky J.C., Mack S., Fonseca B.D., Das S.R., Berman A.J., Durrant J.D. (2019). Capturing the Mechanism Underlying TOP mRNA Binding to LARP1. Structure.

[55] Kozlov G., Mattijssen S., Jiang J., Nyandwi S., Sprules T., Iben J.R., Coon S.L., Gaidamakov S., Noronha A.M., Wilds C.J. (2022). Structural basis of 3′-end poly(A) RNA recognition by LARP1. Nucleic Acids Res..

[56] Wei N., Deng X.W. (2003). The COP9 signalosome. Annu. Rev. Cell Dev. Biol..

[57] Chamovitz D.A., Segal D. (2001). JAB1/CSN5 and the COP9 signalosome: A complex situation. EMBO Rep..

[58] Yuan C., Wang D., Liu G., Pan Y. (2021). Jab1/Cops5: A promising target for cancer diagnosis and therapy. Int. J. Clin. Oncol..

[59] Sussman M.A., Völkers M., Fischer K., Bailey B., Cottage C.T., Din S., Gude N., Avitabile D., Alvarez R., Sundararaman B. (2011). Myocardial AKT: The Omnipresent Nexus. Physiol. Rev..

[60] Latronico M.V., Costinean S., Lavitrano M.L., Peschle C., Condorelli G. (2004). Regulation of cell size and contractile function by AKT in cardiomyocytes. Ann. N. Y. Acad. Sci..

[61] Hilfiker-Kleiner D., Haghikia A., Berliner D., Vogel-Claussen J., Schwab J., Franke A., Schwarzkopf M., Ehlermann P., Pfister R., Michels G. (2017). Bromocriptine for the treatment of peripartum cardiomyopathy: A multicentre randomized study. Eur. Heart J..

[62] Attachaipanich T., Attachaipanich S., Kaewboot K. (2025). Efficacy and safety of bromocriptine in peripartum cardiomyopathy: A systematic review and meta-analysis. Int. J. Cardiol..

[63] Morales A., Painter T., Li R., Siegfried J.D., Li D., Norton N., Hershberger R.E. (2010). Rare variant mutations in pregnancy-associated or peripartum cardiomyopathy. Circulation.

