# Supplementary figures and images for: Transcriptome Analysis Identifies Proteostasis and Cell Survival Pathway Disruption in Peripartum Cardiomyopathy, Leading to Heart Failure
Source: Cells. 2026 Apr 15;15(8):698. doi: 10.3390/cells15080698 (PMC13114936; doi:10.3390/cells15080698)

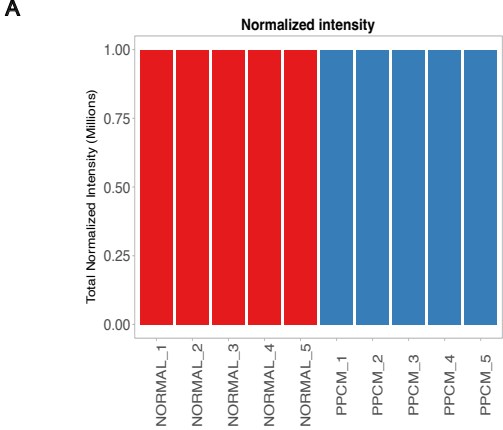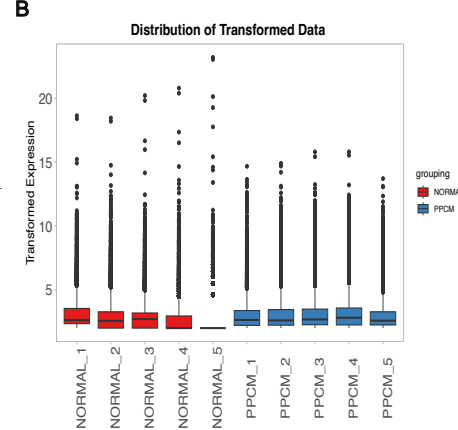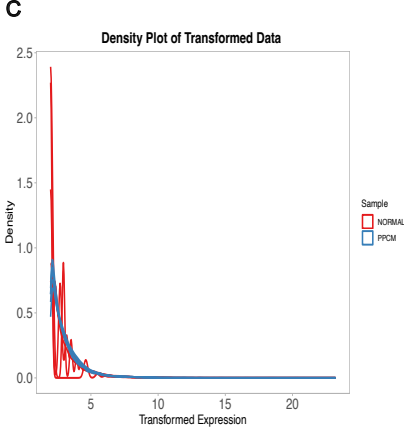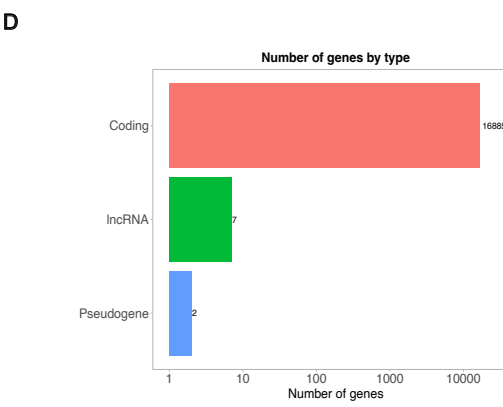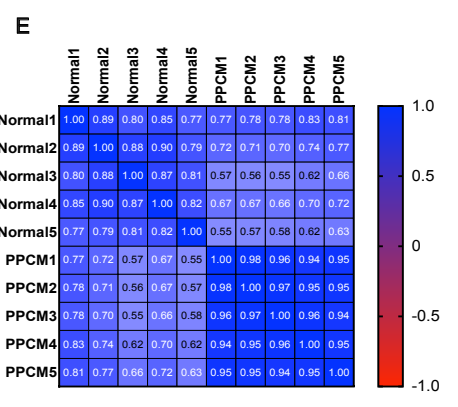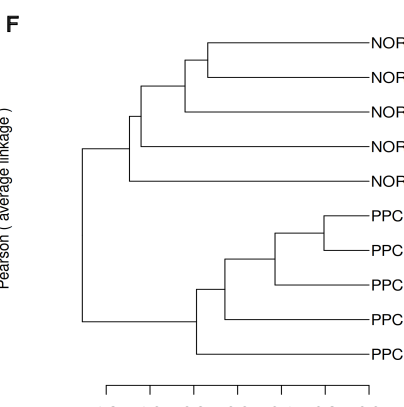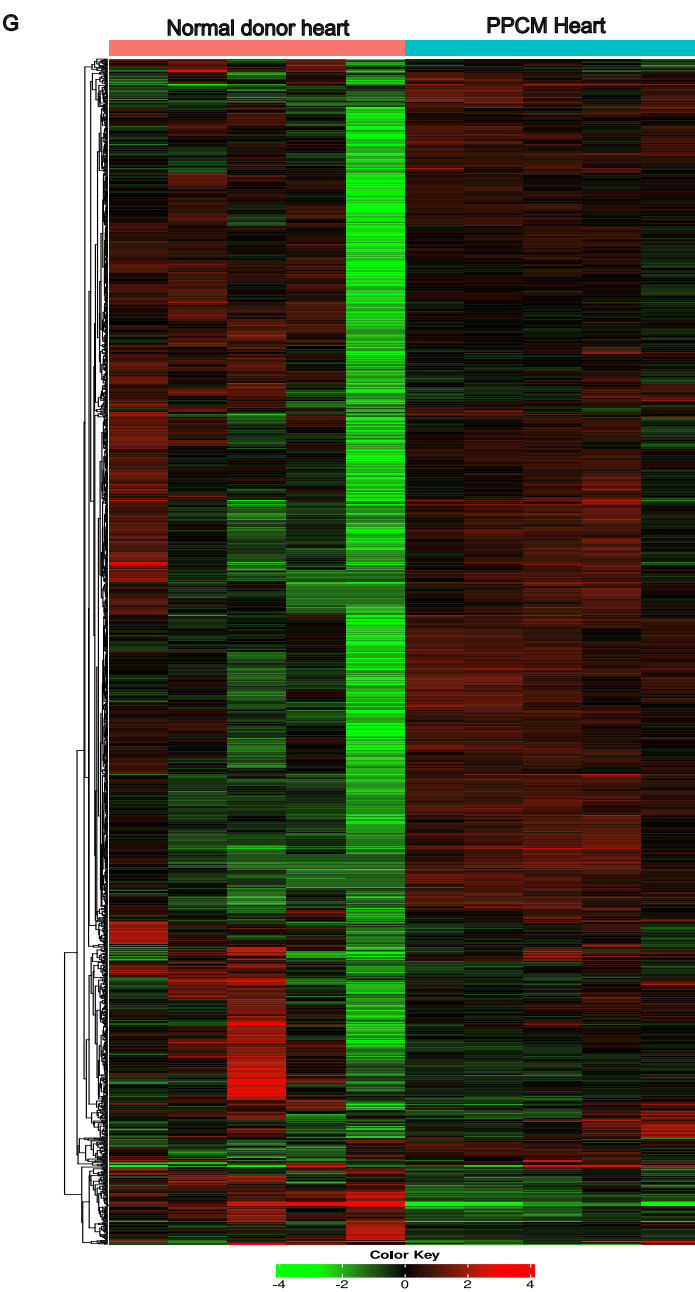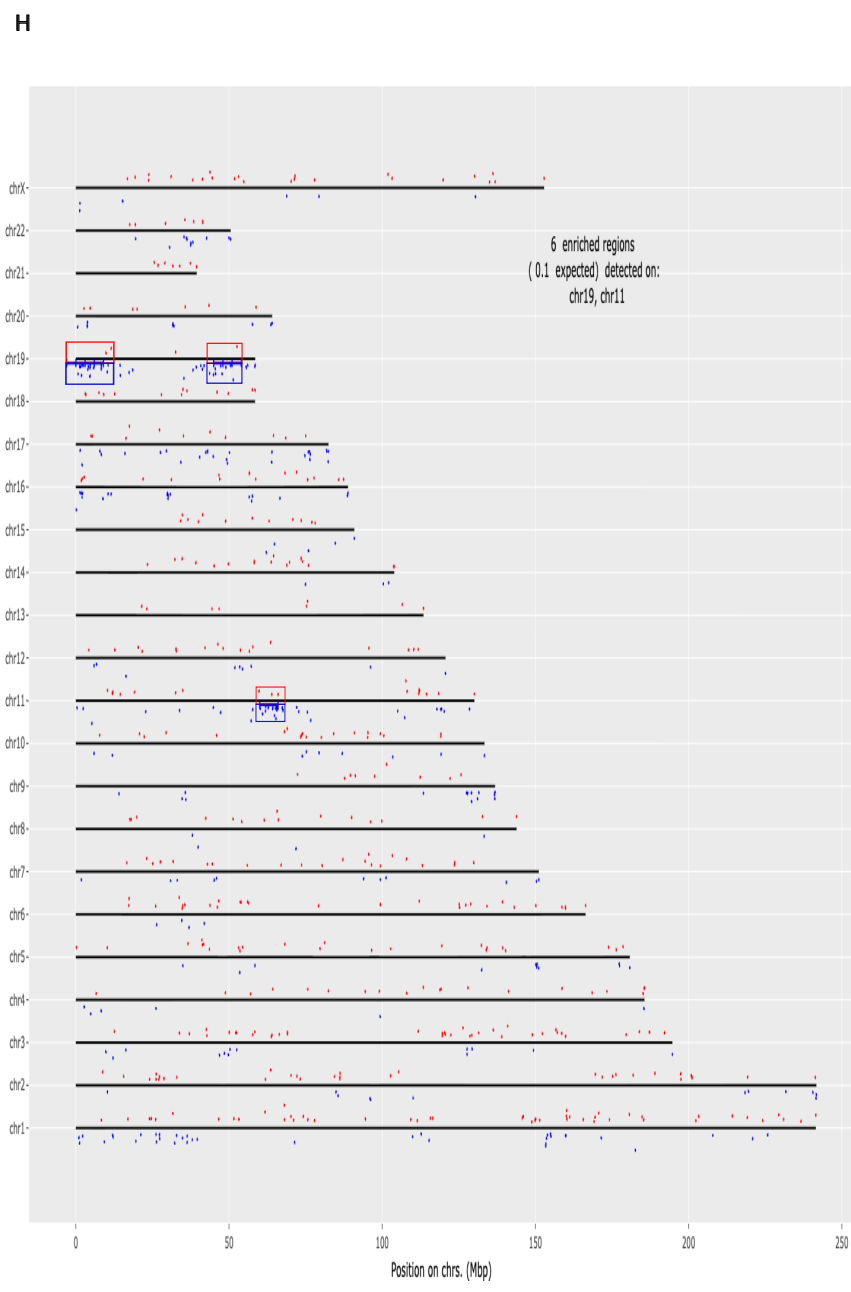

Supplement: Supplementary file 1 [file cells-15-00698-s001.zip › Supplementary Figure S1.pdf]

A

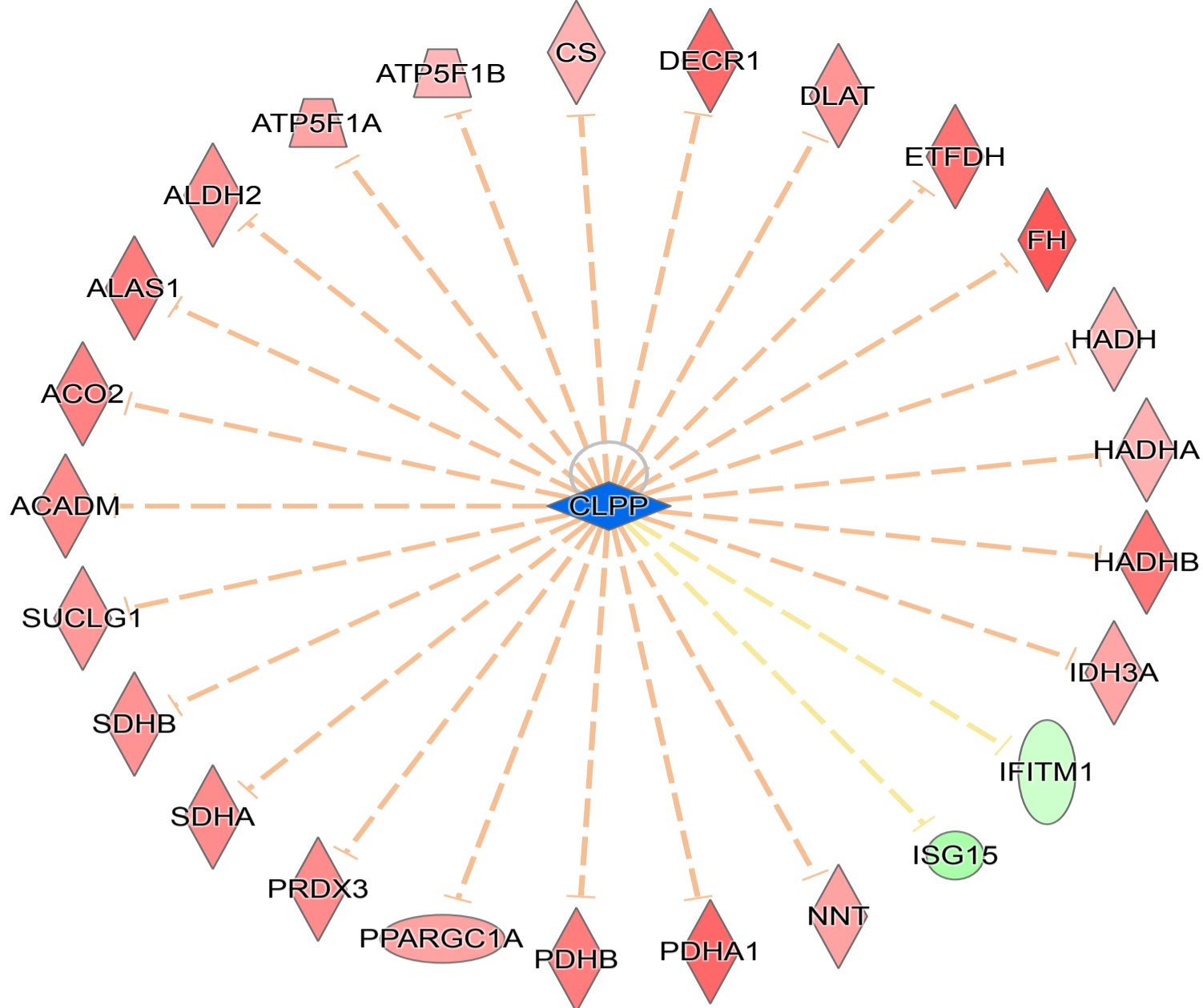

B

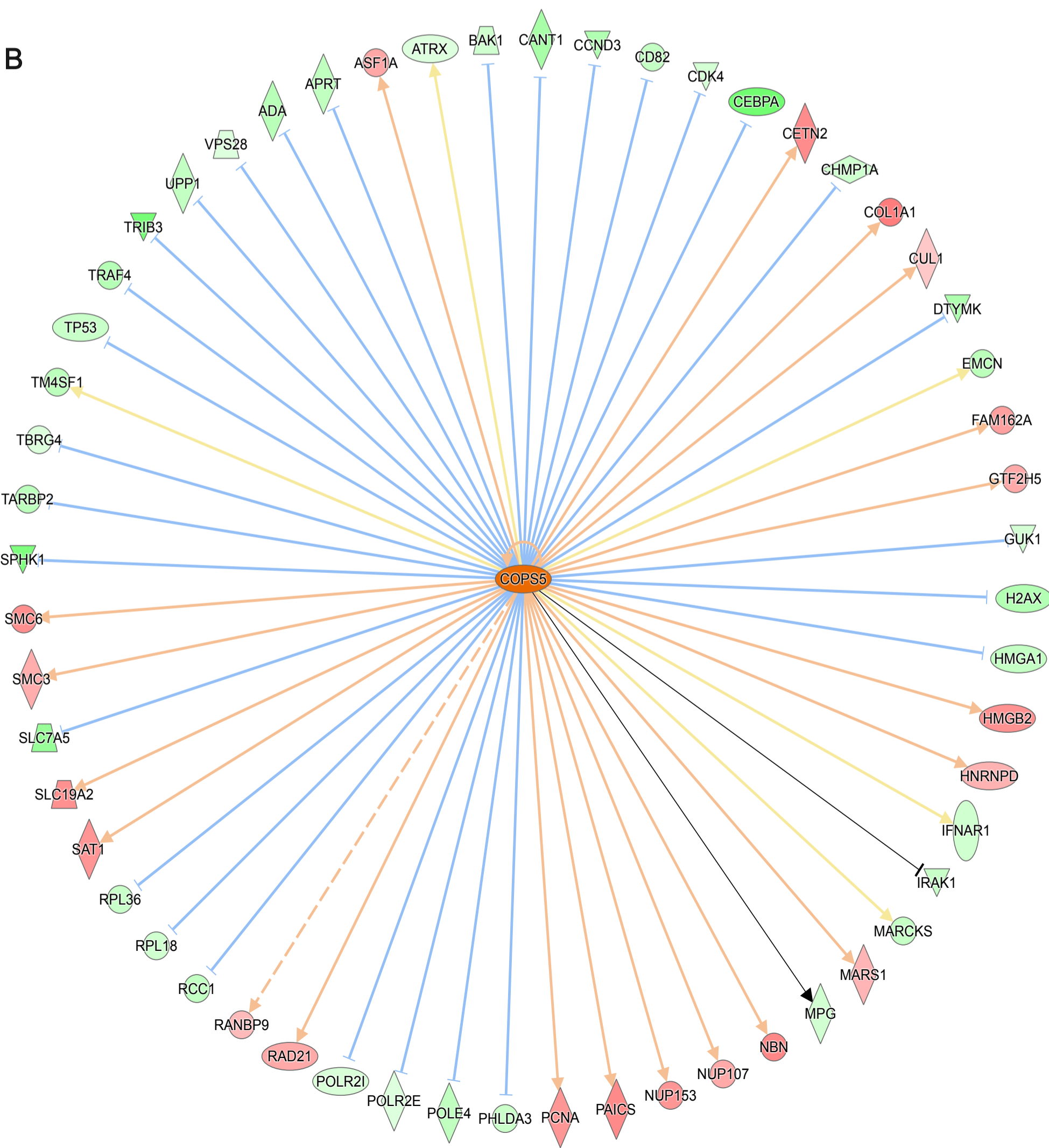

Supplement: Supplementary file 1 [file cells-15-00698-s001.zip › Supplementary Figure S2.pdf]
